# Neuronal Cellular Responses to Extremely Low Frequency Electromagnetic Field Exposure: Implications Regarding Oxidative Stress and Neurodegeneration

**DOI:** 10.1371/journal.pone.0104973

**Published:** 2014-08-15

**Authors:** Marcella Reale, Mohammad A. Kamal, Antonia Patruno, Erica Costantini, Chiara D'Angelo, Miko Pesce, Nigel H. Greig

**Affiliations:** 1 Department of Experimental and Clinical Sciences, University “G. d'Annunzio, Chieti, Italy; 2 King Fahd Medical Research Center, King Abdulaziz University, Jeddah, Kingdom of Saudi Arabia; 3 Department of Medicine and Aging Science, University 'G. d'Annunzio' of Chieti-Pescara, Chieti, Italy; 4 Drug Design and Development Section, Translational Gerontology Branch, Intramural Research Program, National Institute on Aging, National Institutes of Health, Baltimore, Maryland, United States of America; National Insttitute on Drug Abuse, United States of America

## Abstract

Neurodegenerative diseases comprise both hereditary and sporadic conditions characterized by an identifying progressive nervous system dysfunction and distinctive neuopathophysiology. The majority are of non-familial etiology and hence environmental factors and lifestyle play key roles in their pathogenesis. The extensive use of and ever increasing worldwide demand for electricity has stimulated societal and scientific interest on the environmental exposure to low frequency electromagnetic fields (EMFs) on human health. Epidemiological studies suggest a positive association between 50/60-Hz power transmission fields and leukemia or lymphoma development. Consequent to the association between EMFs and induction of oxidative stress, concerns relating to development of neurodegenerative diseases, such as Alzheimer disease (AD), have been voiced as the brain consumes the greatest fraction of oxygen and is particularly vulnerable to oxidative stress. Exposure to extremely low frequency (ELF)-EMFs are reported to alter animal behavior and modulate biological variables, including gene expression, regulation of cell survival, promotion of cellular differentiation, and changes in cerebral blood flow in aged AD transgenic mice. Alterations in inflammatory responses have also been reported, but how these actions impact human health remains unknown. We hence evaluated the effects of an electromagnetic wave (magnetic field intensity 1mT; frequency, 50-Hz) on a well-characterized immortalized neuronal cell model, human SH-SY5Y cells. ELF-EMF exposure elevated the expession of NOS and O_2_^−^, which were countered by compensatory changes in antioxidant catylase (CAT) activity and enzymatic kinetic parameters related to CYP-450 and CAT activity. Actions of ELF-EMFs on cytokine gene expression were additionally evaluated and found rapidly modified. Confronted with co-exposure to H_2_O_2_-induced oxidative stress, ELF-EMF proved not as well counteracted and resulted in a decline in CAT activity and a rise in O_2_^−^ levels. Together these studies support the further evaluation of ELF-EMF exposure in cellular and in vivo preclinical models to define mechanisms potentially impacted in humans.

## Introduction

Neurodegenerative diseases are characterized by a slow and progressive loss of neurons within the central nervous system (CNS). They generally occur in later life, are often associated with deficits in brain function (e.g., memory and cognition, or movement – depending on the predominant neuronal population impacted) and are defined as hereditary and sporadic conditions; with the majority of being non-familial [1]–[3]. There are hundreds of disorders that could be described as neurodegenerative diseases. Many are rare, a few are common and include Alzheimer's disease (AD), Parkinson's disease (PD), amyotrophic lateral sclerosis (ALS), multiple sclerosis, Huntington's disease (HD) and multiple system atrophy (MSA), and these, particularly when combined together, represent one of the most critical health concerns currently impacting developed countries. Neurodegenerative diseases often extend over a decade prior to death, but the actual onset of neurodegeneration may silently precede clinical manifestations by numerous years; for AD by as much as two or three decades [4], [5]. Specific environmental factors and lifestyle are considered to play a key role in the pathogenesis of neurodegenerative disorders [6]–[8]. These can occur during early life and remain quiescent [9], [10].

Previous studies have reported that exposure to extremely low-frequency electromagnetic fields (ELF-EMF) can alter animal behavior, cerebral blood flow in aged AD transgenic mice, and modulate gene expression, cell differentiation and survival of neural cell populations [11]–[13]. An elevated risk of neurodegenerative diseases has been reported in some subjects with occupational exposure to ELF-EMF at magnetic field levels comparable with those present in some residential areas (0.2–5.0 µT). In general, however, epidemiological studies have largely failed to find strong positive associations between neurodegenerative disease occurrence and EMF exposure [14], [15]. This could be due to multiple issues that include (i) the wide variability of exposure levels between individuals, (ii) the heterogeneic characteristics and small number of subjects studied, (iii) the intensity and time of EMF exposure, (iv) the target cell phenotype evaluated and, in particular, (v) the selection and appropriateness of endpoints appraised. In light of these considerations, there is strong rationale to evaluate mechanisms via which EMFs may impact neuronal processes to focus epidemiological studies and support the selection of defined future endpoints.

Albeit that human data bears the most direct relevance to human disease, its interpretation is often difficult. Developing and testing hypotheses is generally more easily undertaken in cellular studies. Additionally, their much reduced cost and relative ease of manipulation can allow evaluation of the role of specific environmental factors, either alone or in combination with other agents, on key cellular targets considered central to neurodegenerative disease development. In relation to molecular events potentially impacted by ELF-EMF, live, adult human neurons are not readily available. However, as a valuable alternative, human SH-SY5Y cells express a neuronal phenotype and represent a well characterized immortalized line that, although removed from the *in vivo* system, provide the opportunity to study responses in human rather than rodent neural cells [15].

A large body of evidence supports a direct contribution of inflammation in the development and progression of neurodegeneration [16]. In this regard, a wide range of inflammatory markers, either absent or minimally expressed in the healthly population, have been found present in AD, MS, PD, HD, ALS and MSA [17]–[22]. Additionally, oxidative stress, marked by lipid peroxidation, nitration, reactive carbonyls, and nucleic acid oxidation, is perhaps the earliest feature of neurodegeneration [23], [24] and occurs in vulnerable neurons preceding any defining classical pathology.

Within all aerobic cells, and particularly for highly metabolic neurons, the processes involved in energy production and respiration inevitably generate reactive oxiygen and nitrogen species (ROS and RNS, respectively), which represent a wide range of small signaling molecules with highly reactive unpaired valence electrons. Oxidative stress occurs when ROS/RNS production exceeds the abilities of resident antioxidant defense mechanisms to sequester free radical intermediates, which consequently escape to then damage major macromolecules [23], [24]. For example, the presence of the reactive peroxynitrite has been observed in acute and chronic active MS lesions, and nitric oxide metabolites, lipid peroxidation products are reported significantly elevated in the serum of patients with MS [25]. A pronounced increase in NO levels has been described in ALS [26], and oxidative stress is a common downstream mechanism by which nigral dopamine neurons are damaged in PD [27]. ROS is additionally generated by the activation of several inflammatory enzymes, for example the expression of inducible nitric oxide synthase (iNOS) is under the transcriptional control of a variety of inflammatory cytokines, and the expression of proinflammatory mediators appears to be redox sensitive [28],[29].

The fine control of inflammatory mediator levels appears to be critical to neuronal homeostasis and health. As an example, a deficiency in neuronal TGF-β signaling promotes neurodegeneration and AD [30], whereas augmented TGF-β can act as an anti-inflammatory cytokine and has potential neuroprotective action in AD and following a CNS insult [31], [32]. Interleukin 18 (IL-18) exerts proinflammatory effects by inducing gene expression and synthesis of cytokines, chemokines and adhesion molecules, whereas its natural inhibitor, IL-18 binding protein (Il-1BP), operates as a key negative feedback mechanism to both balance and limit the impact of inflammation [33]. Likewise, the actions of microglial cells are regulated at a number of stages, such as at the level of their movement by the monocyte chemoattractant protein (CCL2/MCP-1) [34]–[36].

In the current study, we investigated the impact of an electromagnetic wave (magnetic field intensity, 1mT; frequency, 50 Hz) on SH-SY5Y cell cultures in relation to oxidative stress, with a focus on select mechanisms to balance oxidative damage and inflammation. This well characterized cellular model possesses a number of physiological systems that have parallels to human neurons to provide potential insight into cellular cascades that, by exposure to ELF-EMF, may lead towards neurodegeneration.

## Materials And Methods

### Cell culture

The neuroblastoma cell line, SH-SY5Y (Sigma-Aldrich, St Louis, MO, USA), was grown in Dulbecco's modified Eagle's medium (DMEM) containing 10% foetal bovine serum and a mixture of streptomycin/penicillin. Cultures were maintained at 37°C in a humidified atmosphere of 5% CO_2_. On a weekly basis, SH-SY5Y cells were detached and seeded into dishes or multiwell plates and, on reaching 60–70% confluency, cells were continuously exposed to a 50-Hz ELF-EMF at a flux density of 1 mT (r.m.s.) produced by an electromagnetic generator located on a grid to allow air circulation within the central part of its solenoid. Control, non-exposed cell cultures were grown simultaneously. Specifically, cells were placed in a different incubator and cultured under the same conditions and times as ELF-EMF exposed cells. At the end of incubation, both exposed and non-exposed cells were harvested using trypsin-EDTA. Their viability was evaluated by Trypan blue dye exclusion and they were counted in a Burker chamber. In preliminary studies responses to ELF-EMF appeared independent of cell density in challenged cells, and cell density remained similar between challenged and unchallenged cells.

### Magnetic field exposure system and exposure conditions of cell cultures

The experimental setup and ELF-EMF exposure system have been previously described [37]. Briefly, an oscillating magnetic field (AC MF) consisted of: (i) a sinusoidal signal 50 Hz waveform generator (Agilent Technologies model 33220A, Santa Clara, CA, USA); (ii) a power amplifier (NAD electronics Ltd., model 216, London, UK); (iii) an oscilloscope (ISO-TECH model ISR658, Vicenza, Italy) dedicated to monitoring output signals from a Gaussmeter (MG-3D, Walker Scientific Inc., Worcester, MA, USA) and the AC MF generator; (iv) a 160 turn solenoid (22 cm length, 6 cm radius, copper wire diameter of 1.25×10^−5^ cm) that generated a horizontal magnetic field. This solenoid was placed inside the exposure incubator. The achieved MF intensity (1 mT (rms)) was continuously monitored using a Hall-effect probe connected to the Gaussmeter. The environmental magnetic noise inside the incubator was related to the geomagnetic field (≈40 µT) and to the 50 Hz disturbance associated with the working incubator (≈7 µT (rms).

Cell cultures were maintained in a 5% CO_2_ atmosphere and at a temperature of 37±0.3°C. For ELF-EMF challenge, cells were placed within the central region of the solenoid that was characterized by the greatest field homogeneity (98%) to thereby receive continuous exposure to steady-state 50-Hz ELF-EMF for defined times up to 24 hr; thereafter, cell were immediately harvested for the outcome analyses described below. In addition, a further digital thermometer (HD 2107.2, Delta OHM, Padova, Italy) was placed inside the solenoid directly alongside the cell cultures to record local temperature variations, and the temperature of the cell medium was recorded using a specially designed thermoresistor (HD 9216; Delta OHM, Padova, Italy). No significant temperature changes were observed associated with application of the ELF field (ΔT = 0.1°C). This lack of a thermal effect on cells maintained at 37°C is in accord with the known non-thermal nature of ELF interactions with biological molecules [15], [37]. Any low-level Joule heating was efficiently dissipated by the incubator's fan mechanism as Dt was <0.1308°C in the medium of exposed cells.

### Analyses of NOS activity

At defined times, ELF-EMF exposed and unexposed (control) cells were immediately analysed for NOS activity. This was assayed by measuring the conversion of L-[2,3-^3^H]arginine to L-[2,3-^3^H]citrulline in HaCaT cell homogenates. Briefly, 1 µl of radioactive arginine, L-(2,3,4,5)-[^3^H]Arginine Monohydrochloride 64 Ci/mM, 1 µCi/ µl (Amersham, Arlington Heigths, IL, USA), 5 µl NADPH 10 mM, and 5 µl CaCl_2_ 6 mM (Calbiochem, San Diego, CA, USA) were added to each sample, which then were incubated for 30 min at room temperature. Thereafter, the reaction was stopped by the addition of 400 µl stop-buffer (50 mM HEPES, pH 5.5, and 5 mM EDTA). Unreacted arginine was removed by the addition of equilibrated cationic exchange resin (Dowex AG50WX-8, Sigma-Aldrich). After centrifugation, the radioactivity, corresponding to L-[^3^H]-citrulline in the eluate, was measured by liquid scintillation spectrometry and expressed as pmol^3^H/min/mg.

### Determination of O_2_^−^

Production of O_2_^−^ was determined spectrophotometrically (Hewlett Packard 8452 A, Palo Alto, CA, USA) by monitoring the reduction of ferricytochrome c (Type VI, Sigma-Aldrich) at 550 nm, as described by Pritchard and colleagues [38]. Briefly, ferricytochrome *c* (50 µmol/L) was added to cuvettes containing cells and PBS (final volume 1 mL), either in the presence or absence of superoxide dismutase (SOD, 350 U/ml) and subsequent changes in absorbance were followed for 10 min. Rates of O_2_^−^ production were calculated on the basis of the molar extinction coefficient of the reduced ferricytochrome c [e = 21000 cm ^−1^ (mol/L) ^−1^]. Cell counts were used to calculate results as nmol O_2_^−^/10^6^ cells/min.

### Measurement of catalase (CAT) activity

CAT activity was measured spectrophotometrically as previously described [39]. The decomposition of H_2_O_2_ was monitored continuously at 240 nm. The assay mixture, in a final volume of 3 ml, contained 10 mM potassium phosphate buffer, 10 mM H_2_O_2_ and 10 µg of protein enzymatic extract. CAT units were defined as 1 µmole H_2_O_2_ decomposed/min at 25°C.

### RNA extraction and RT-PCR analysis

Total RNA was extracted from SH-SY5Y cell cultures using TRIzol reagent (Invitrogen, Life Technologies, Paisley, UK) according to the manufacturer's protocol. The RNA concentration was estimated by measuring its absorbance at 260 nm using a Bio-Photometer (Eppendorf, Milano, Italy), and RNA samples were kept frozen at −80°C until use. Purified RNA was electrophoresed on a 1% agarose gel to assess the integrity of the purified RNA. One μg of RNA was reverse transcribed into cDNA using a Quantitect reverse transcription kit (Qiagen, Milano, Italy), according to the manufacturer's instructions. Polymerase chain reaction (PCR) was performed using the mRNA/cDNA specific cytokine primer pairs (Table 1). All PCR reactions were performed in a PCR-thermocycler (Eppendorf, Milano, Italy). The program utilized for PCR amplification was as follows: an initial period of 5 min at 95°C, followed by a variable number of cycles of 30 s denaturation at 95°C, 30 s annealing at 60°C and finally 30 s of extention at 72°C. The programme was terminated with a period of 10 min at 72°C. To be within the exponential phase of the semi-quantitative PCR reaction, the appropriate number of cycles was newly established for every set of samples. Products were separated by gel electrophoresis on 2% agarose gels and visualized by ethidium bromide staining. All gels were scanned and the percent adjusted volume intensities of all of the RT-PCR products were determined using a Bio-Rad gel documentation system (Bio-Rad, Hercules, CA, USA).). Mean ± SD intensities were calculated for all PCR experiments.

**Table 1:** Sequences of the oligonucleotide primers utilized in the reverse transcriptase polymerase chain reaction.

Gene	Forward Primer Sequence [5'-3']	Reverse Primer Sequence [5'-3']	Primer size
RPS18	CTTTGCCATCACTGCCATTAAG	TCCATCCTTTACATCCTTCTGTC	199 bp
IL18	CAGTCAGCAAGGAATTGTCTC	GAGGAAGCGATCTGGAAGG	139 bp
IL18BP	CAACTGGACACCAGACCTCA	AGCTCAGCGTTCCATTCAGT	235 bp
TGFβ1	AACAATTCCTGGCGATACCTC	GTAGTGAACCCGTTGATGTCC	197 bp
MCP1	AACTGAAGCTCGCACTCTCG	GAGTGAGTGTTCAAGTCTTCGG	327 bp

### Cell viability

Cell viability was determined by quantitative colorimetric MTT assay (Sigma-Aldrich) using 96-well microplates immediately following ELF-EMF and control (no exposure) challenge. Briefly, 50 ul of the MTT-labeling reagent, at a final concentration of 0.5 mg/ml, was added to each well at the end of the dexamethasone/folic acid period, and the plate was then placed in a humidified incubator (37°C, and 5% CO2 and 95% air (v/v)) for an additional 2 hr period. Thereafter, the production of formazan was read at 540 nm λ using a standard 96-well plate reader. The intensity of the color produced was proportional to the number of living SH-SY5Y cells.

### Statistical analysis

Band intensities of RT- PCR were quantified by a densitometer and expressed as relative values to the controls. Data are expressed as means ± SD from three or more independent experiments. For statistical analysis, quantitative data were analyzed by Student t test, with appropriate corrections in the case of multiple comparisons. Differences are considered significant at p<0.05.

## Results

### ELF-EMF does not impact cell morphology and viability

**Figure 1:**
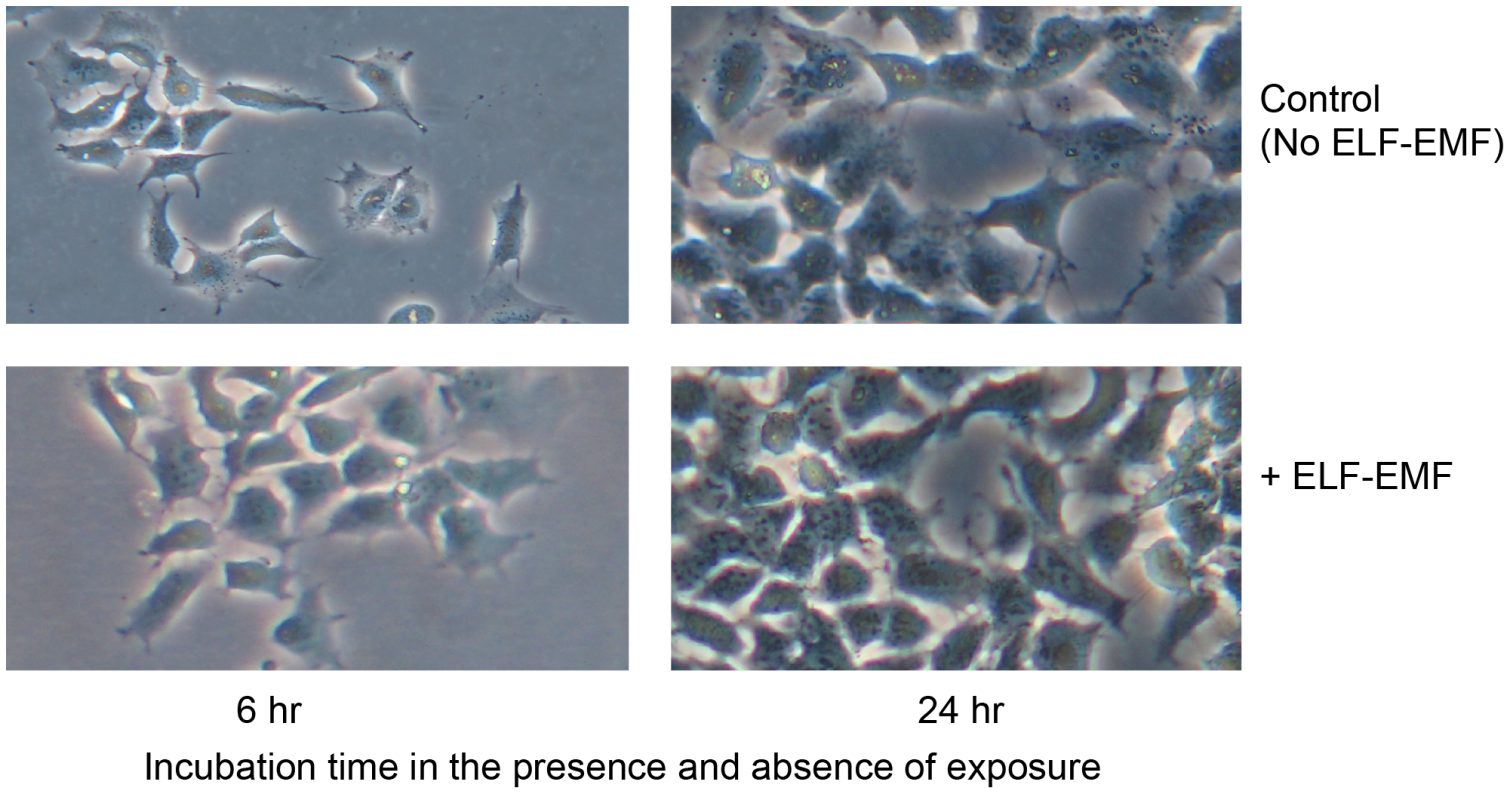
ELF-EMF (1 mT (rms), 50 Hz) exposure did not impact phenotypic expression, growth or viability of human SH-SY5Y cells. Representative phase contrast images of proliferative SH-SY5Y cells in culture medium with 10% FBS (400× magnification), grown for 6 and 24 hr the presence and absence of ELF-EMFs. Not shown, cell proliferation was not influenced by the presence of ELF-EMF, as determined by MTT assay at 6 and 24 hr.

Human SH-SY5Y cultures were subjected to ELF-EMF 50 Hz, 1.0 mT exposure and their cell morphology and proliferation rate were compared to unexposed control cells. Figure 1 illustrates representative images of exposed and control cultures at both 6 and 24 hr. No differences in cell morphology were evident at either time. Additionally, as quantitatively evaluated by MTT assay at 24 hr as well as by image analysis, the cellular proliferation rate was not influenced by the presence of ELF-EMFs; with exposed and unexposed cultures possessing comparable cell numbers.

### ELF-EMF elevates NOS activity

**Figure 2:**
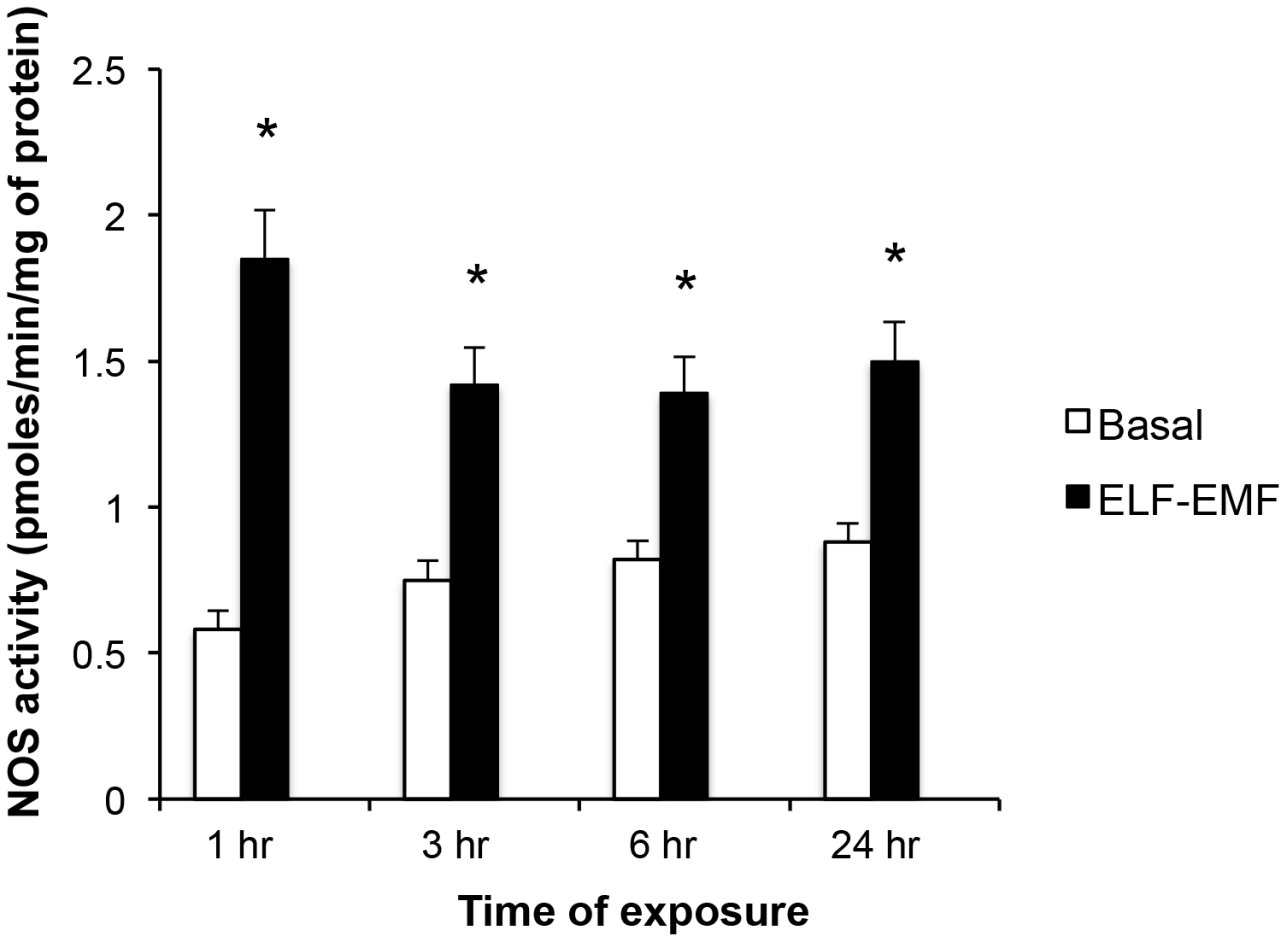
ELF-EMF exposure induced a rapid and sustained elevation in NOS activity. NOS enzymatic activity, determined by measuring the conversion of L-[^3^H]arginine to L-[^3^H]citrulline, is expressed in picomoles per minute per milligram of protein. Each value represents the mean ± s.d. of different experiments performed in triplicate (*p<0.05).

NOS activity, determined by measuring the conversion of L-[2,3-^3^H]arginine to L-[2,3-^3^H]citrulline, was increased in SH-SY5Y cells exposed to ELF-EMF. As shown in Figure 2, this elevation was significant across all times evaluated (1, 3, 6 and 24 hr), and proved greatest at 1 hr (3.2-fold rise) versus later times (3 hr: 1.9-fold, 6 and 24 hr: 1.7-fold elevation).

### Effect of ELF-EMF on antioxidant enzyme catalase (CAT) activity

**Figure 3:**
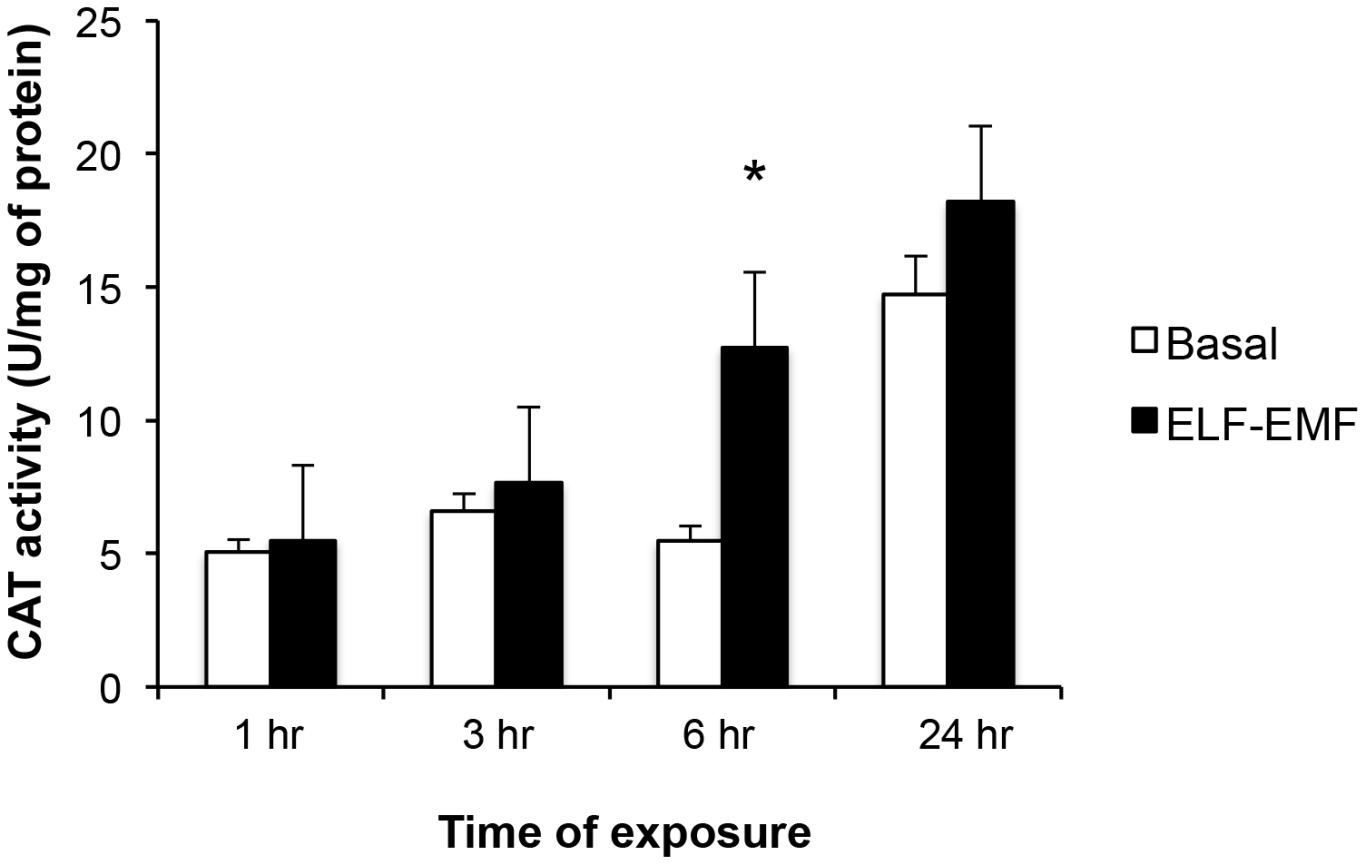
ELF-EMF exposure induced a time-dependent elevation in Catylase (CAT) activity: CAT activity was normalized with respect to the total protein content of cell lysates and expressed as units per mg of protein. Each value was the mean ± s.d. of three independent experiments performed in triplicate (*p<0.05).

The impact of ELF-EMF-exposure on CAT activity in SH-SY5Y cells was evaluated. As illustrated in Figure 3, under basal conditions CAT activity remained largely unaltered over 1, 3 and 6 hr of incubation, but became elevated by 24 h (2.9-fold vs. 1 hr value). By contrast, the exposure of SH-SY5Y cells to ELF-EMF resulted in significantly heightened CAT activity levels by 6 hr (2.3-fold vs. both the basal level at 6 hr, and the 1 hr level post ELF-EMF exposure).

Table 2 provides key parameters characterizing the kinetics of CAT activity in SH-SY5Y cells exposed and unexposed to ELF-EMF. Exposure induced a modest increase (23%) in total velocity υ of CAT enzyme that was accompanied by a moderate (2.2-fold) rise in the υ_min_ value. The rate of decrease (↓®) in the CAT reaction was found to be elevated in the presence of ELF-EMF (3.3-fold), and the time at which maximum activity was recorded (pT) in response to ELF-EMF exposure occurred at 16.5 hr compared to 13.5 hr for controls (Table 2).

**Table 2:** Kinetic constants characterizing the impact of ELF-EMF exposure on antioxidant enzyme catalase (CAT) activity in human SH-SY5Y neuronal cultures.

	Total υ	↑®	υ_min_	↓®	pT (hr)	pυ
Basal	467±6.0	2.0±0.32	1.7±1.3	2.45	13.5	31.2
+ ELF-EMF	575±8.7	1.5±0.12	3.7±0.49	8.0	16.5	26.2

υ = activity; ↑® =  Upward υ rate; υ_min_ =  minimum initial activity; ↓® =  downward υ rate; pT = peak time; pυ = peak activity.

### Effect of ELF-EMF on cytochrome P450 (CYP-450) activity

**Figure 4:**
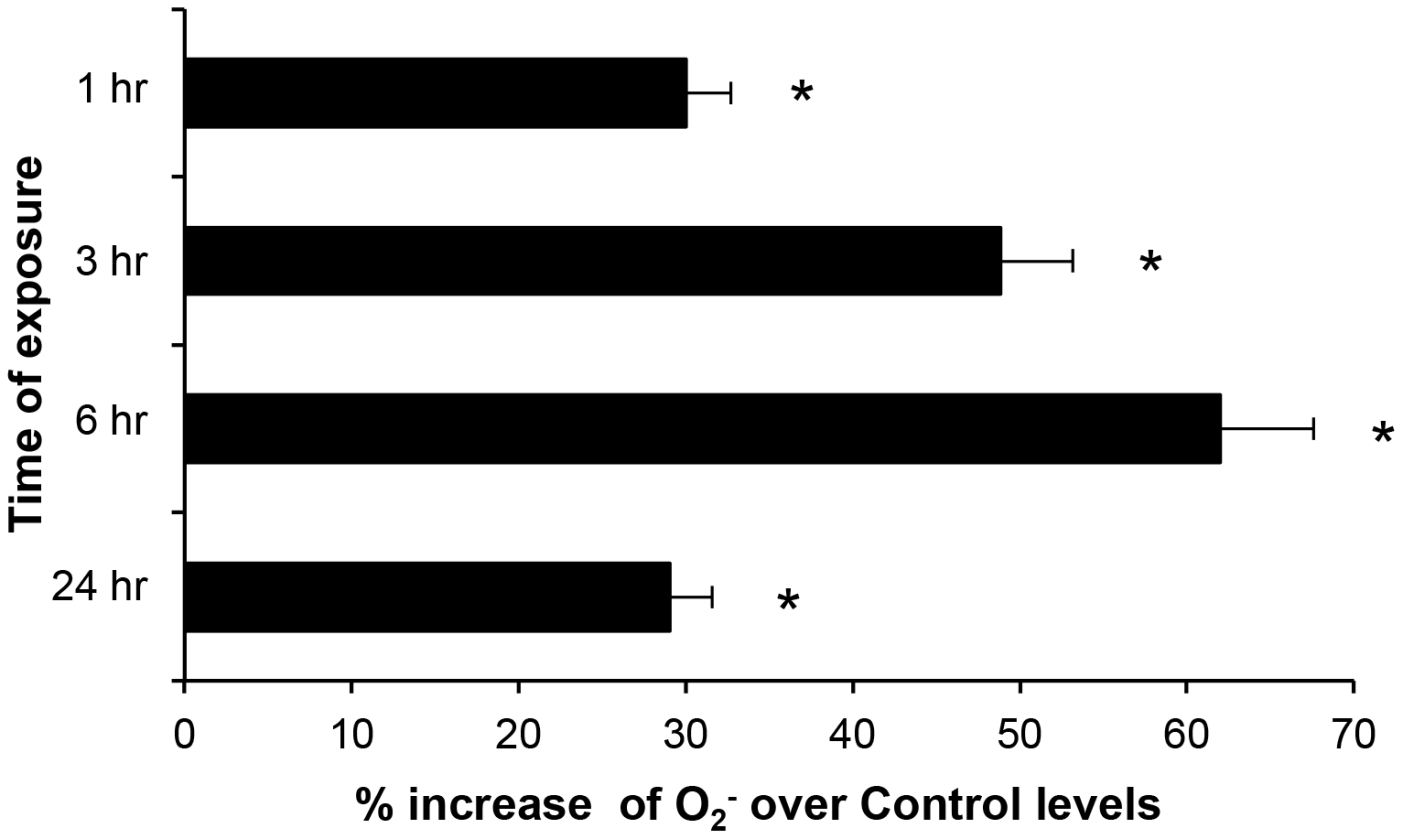
ELF-EMF exposure time-dependently increased O_2_^−^ production: O_2_^−^ production in ELF-EMF exposed SH-SY5Y cells was evaluated by the cytochrome c reduction assay method and expressed as % increase with respect to non exposed SH-SY5Y cells (*p<0.05).

Oxidative stress appears to a primary factor contributing to neuronal dysfunction [23], [40], [52]. As the levels of O_2_^−^ production represents an index of free radical and ROS production, we evaluated the cellular redox state by NADPH oxidase activity using the cytochrome c reduction assay method. Assessment of the effect of ELF-EMF-exposure on CYO 450 activity in SH-SY5Y cells showed that ELF-EMF increased CYP 450 activity and, consequently, O_2_^−^ production at all times studied (Fig. 4), with the greatest increase (62%) occurring after 6 hr of exposure.

A rise (21%) in the total velocity of CYP-450 was measured in response to ELF-EMF exposure, which was accompanied by a significant elevation in the rate of increase (↑®) of the enzymatic reaction by 2.4-fold (Table 3). ELF-EMFS exposure did not significantly impact the υ_min_ value. However, the rate of decrease (↓®) in CYP-450 enzymatic activity was heightened (5.3-fold) in response to ELF-EMF exposure. The time required for CYP-450 activity to peak was shorter, 15.8 vs. 18.8 hr in ELF-EMF exposed and unexposed cultures, respectively, and this peak activity was elevated from 8.1 to 13.95 (1.7-fold) by exposure (Table 3).

**Table 3:** Kinetic constants characterizing the impact of ELF-EMF exposure on cytochrome P450 (CYP-450) activity in human SH-SY5Y neuronal cultures.

	Total υ	↑®	υ_min_	↓®	pT (hr)	pυ
Basal	287.8±14.2	0.34±0.05	3.3±0.20	0.096	18.8	8.1
+ ELF-EMF	348.8±10	0.80±0.097	3.9±0.38	0.506	15.8	13.95

υ = activity; ↑® =  Upward υ rate; υ_min_ =  minimum initial activity; ↓® =  downward υ rate; pT = peak time; pυ = peak activity.

### Effect of ELF-EMF exposure on H_2_O_2_-treated SH-SY5Y cells line

**Figure 5:**
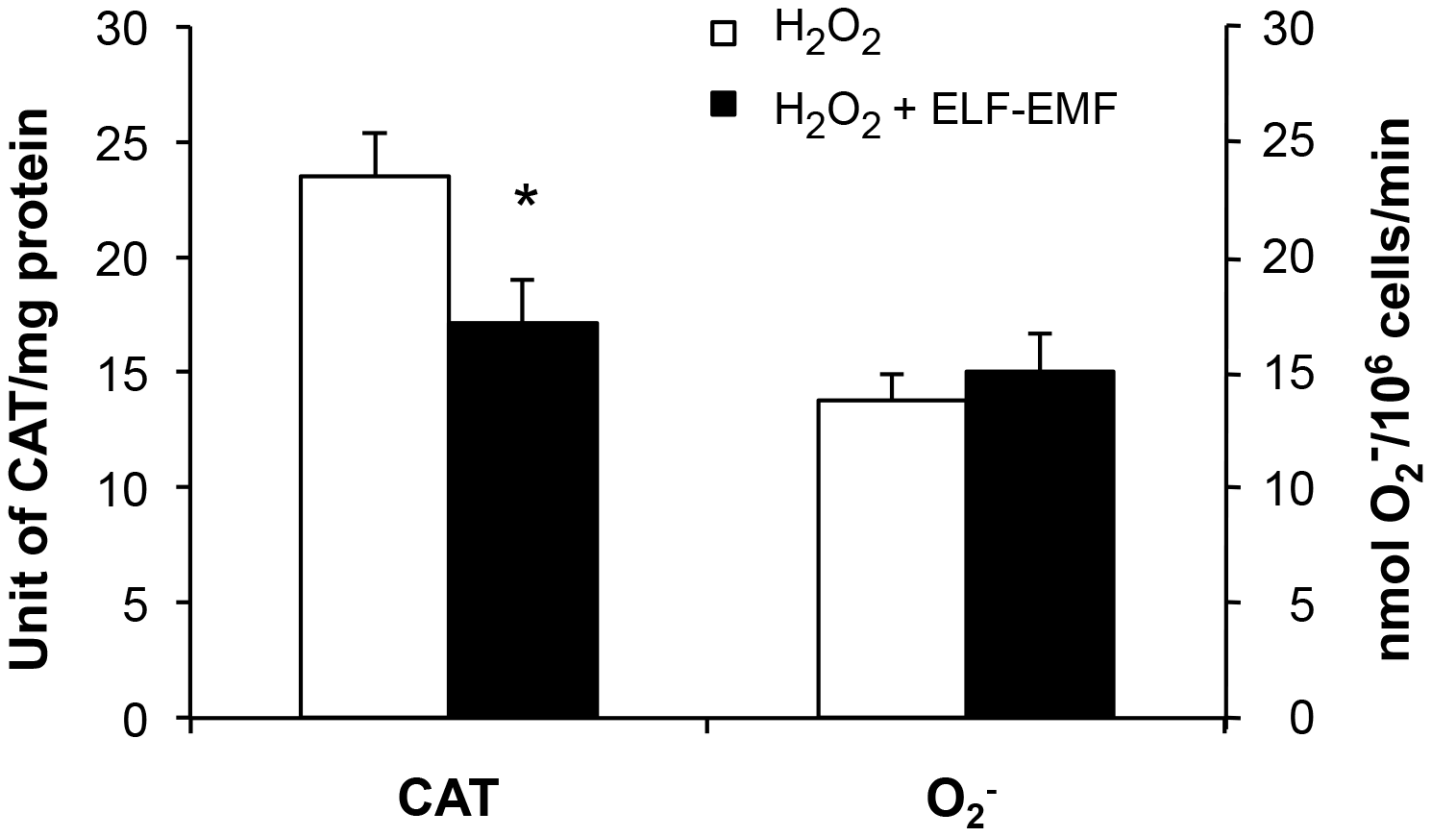
ELF-EMF exposure in the presence of coincident oxidative stress induced a decline in CAT activity. Both CAT activity (normalized to total protein content of cell lysates and expressed as units per mg of protein) and O_2_^−^ production (evaluated by the cytochrome c reduction assay and expressed as nmol O_2_^−^/10^6^ cells/min) were quantified in the presence and absence of pre-existing oxidative stress (100 µM H_2_O_2_) at 24 hr. Each value was the mean ± s.d. of three independent experiments performed in triplicate (*p<0.05).

In light of excessive production of ROS resulting in neuronal damage [23], [24], initial blockade of its generation may prove protective for neurons [40]. To explore whether ELF-EMF may impact the homeostatic capability of neural cells, SH-SY5Y cultures were exposed to H_2_O_2_ in the presence and absence of ELF-EMF. An initial dose-response evaluation of H_2_O_2_ on SH-SY5Y cell viability was performed to identify a subtoxic concentration that would not induce cell death. From this 100 µM H_2_O_2_ was then used in subsequent experiments. This H_2_O_2_ challenge induced a significant rise in CAT activity and O_2_- production in SH-SY5Y cells over a 24 hr incubation period (p<0.05 versus control cells not subjected to H_2_O_2_ (not shown)). As illustrated in Figure 5, co-exposure of cells to ELF-EMF and H_2_O_2_ induced a decline in CAT activity per unit protein (26% reduction (p<0.05) together with a modest rise in O_2_- (9% rise (trend)) with respect to cells treated with H_2_O_2_ alone (Fig. 5). Hence, ELF-EMF exposure of H_2_O_2_-treated cells may increase the cell oxidative activity.

### Effect of ELF-EMF on expression of cyto/chemokines in SH-SY5Y cells

**Figure 6:**
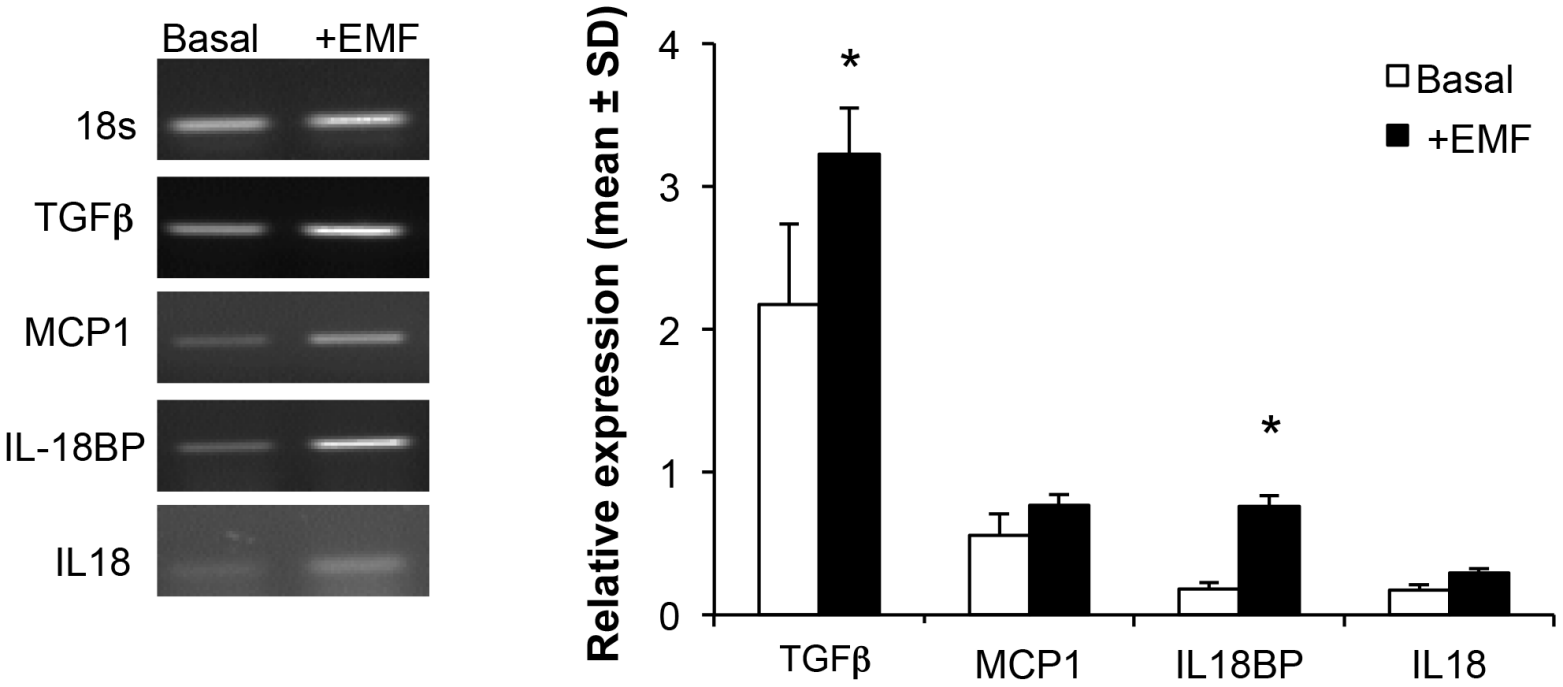
ELF-EMF exposure significantly heightens TGFβ, IL-18 expression. The intensities of bands for TGFβ, IL-18, IL-18BP and MCP-1 are normalized to the GAPDH signals. The expression values are the mean ± S.D. of 2 replicate PCR reactions on RNA isolated from three independent experiments (*p<0.05).

To characterize the impact of ELF-EMF on early ongoing cellular processes, gene transcription of select cyto/chemokines was evaluated in the presence and absence of ELF-EMF exposure. RT-PCR experiments were performed using specific primers and the density of each band was divided by that of its respective β-actin band for normalization, with the resulting value expressed as a relative intensity (Fig. 6). An increase in the expression of TGFβ (1.5-fold, p<0.05) and IL-18BP (4.2-fold, p<0.05) was observed after 24 hr exposure to ELF-EMF, whereas the expressions of IL-18 and MCP-1 were not statistically significantly affected (Fig. 6).

## Discussion

ELF-EMFs are a form of energy, characterized by wavelength or frequency, that are associated with the use of electrical power that generates a magnetic field at a frequency of 50 Hz and a flux density that primarily ranges between 0.2 and 5 mT. The magnetic flux density selected for our study, 1 mT, is 2- and 10-fold the reference levels proposed by the European Community for occupational and general public exposure, respectively. This 1 mT flux density is one of the most studied intensities in medical research focused to elucidate the biological actions of ELF-EMF, consequent to its human translational relevance [41] as during recent years there has been heightened public concern of the impact of ELF-EMFs, associated with both industrial and domesitic use, on human health and welfare. Epidemiological studies have highlighted childhood leukemias, adult primary brain tumors as well as breast cancer, the potential for miscarrage and neurodegenerative disorders [42]–[44]. In large part, past studies have been fraught by limited exposure assessment and other methodological limitations [43], making ELF-EMF epidemiological data interesting but difficult to interpret and then act upon. Whereas the occurrence of some chronic diseases are relatively uncommon (ALS, HD) and have long latency periods for known risk factors, for others – all be they abundant (AD, PD) - their etiologies remain poorly understood [1], [2], which confounds the observation of potential associations, particularly when relying on mortality records [43], [44]. Nevertheless, the World Health Organization [45], [46] and International Programme on Chemical Safety [47] have issued precautions against ELF-EMFs, resulting in exposure level limits being recommended. In parallel with this, there has been increased focus to understand potential mechanisms via which ELF-EMFs may mediate their actions at a cellular level to guide future epidemiological and in vivo research.

The etiology of neurodegenerative diseases is most often multifactorial, and genetic polymorphisms, increasing age as well as environmental cues are primary risk factors [1], [2]. Although different neuronal cell populations are affected across diverse neurodegenerative disorders, hallmark protein modification (whether extracellular amyloid plaques and intracellular neurofibrillary tangles as in AD, or α-synuclein as in PD) is a common feature that supports both differential disease diagnosis and provides a mechanistic basis to gauge disease progression [48], [49].

It is becoming increasingly clear that, particularly for chronic neurodegenerative disorders occurring late in life, a complex combination of risk factors can initiate disease development and modify proteins with physiological functions into ones with pathological roles via a number of defined mechanisms [50]. A common denominator in the occurrence of diverse pathogenic mechanisms is oxidative stress accompanied by redox dysregulation [51], [52], [23], [40], which have a role in metabolic and mitochondrial dysfunction, excitoxicity, calcium handling impairment, glial cell dysfunction and neuroinflammation. Each of these can influence one another at multiple different levels, and hence oxidative stress can both be secondary to them as well as have a primary part in their initiation. Such oxidative stress derives from two primary sources, from chronic ROS creation that is routinely generated from the mitochondrial electron transport chain during normal cellular function [53], and from acute, high levels of ROS generation resulting from nicotinamide adenine dinucleotide phosphate (NADPH) oxidase, particularly associated with the activation of the innate immune system within the CNS [51]. In both circumstances, oxidative stress results when imbalance between ROS production and the clearance of chemically reactive species by endogenous antioxidant enzymes and reducing agents occurs. Environmental factors such as ELF-EMFs, stressors, or disease that augment the former or lower the latter can amplify and drive the process. Thus, in practical terms, oxidative stress is determined by excessive exposure to oxidant molecules when there is insufficient availability of antioxidant mechanisms [23], with the resulting ROS oxidizing vulnerable cellular constituents, including proteins, nucleic acids and lipids, inducing microglial activation, inducing pro-inflammatory and suppressing anti-inflammatory cytokines and related signaling pathways, and ultimately causing both synaptic and neuronal damage and dysfunction. In this regard, the neuronal properties of SH-SY5Y human neuroblastoma cells, together with their pronounced sensitivity to oxidative stress and inflammation, make these cells a valuable model to study a number of neurological pathologies at the molecular, morphological and physiological level.

Previous studies have demonstrated that the cellular effects of ELF-EMFs depend, in large part, on their intensity and exposure time, as well as on the phenotype of the cellular target and interactions with intracellular structures [43], [54]. In SH-SY5Y cell cultures and a number of other cell types exposed to ELF-EMF, genes involved in the stress response, cell growth and differentiation or protein metabolism have been reported to be generally down-regulated, whereas genes involved in Ca^2+^ metabolism, the PI3-kinase pathway, trascription and it's modulation by splicing are up-regulated [41], [54]–[61]. Such actions are reported to often be accompanied by changes in cell growth and oxidative balance [41], [54], [61]. In our experimental conditions, timed continuous ELF-EMF (1 mT (rms)), 50 Hz) exposure likewise impacted cellular oxidative status, causing an early rise in NOS activity and O_2_^−^ levels in SH-SY5Y cells. Within a short duration, this was counteracted by a compensatory increase in the antioxidant capacity of CAT to, thereby, provide the potential to more effectively scavenge any prospective ELF-EMF-mediated ROS over-production; thus avoiding oxidative ELF-EMF-induced cellular damage. In this regard, NOS activity peaked following 1 hr of ELF-EMF exposure and, thereafter, its level declined and remained approximately constant (Fig. 2). Accompanying this, a rise in CAT activity was observed after 6 hr of ELF-EMF exposure (Fig. 3) that was associated with enzyme kinetic changes (Table 2). In light of this, ELF-EMF exposure time-dependently elevated the rate of cellular O_2_^−^ production (Fig. 4), which peaked at 6 hr (162% of control values) and, thereafter, declined toward the baseline value. Hence ELF-EMF exposure can be considered to induce an “activated” cellular state, wherein the enhanced generation and the release of free radicals is offset by a compensatory modulation of antioxidant defences, leading to an absence of negative effects on cell growth and viability.

The kinetic evaluation of the catalyzed antioxidant processes proved useful in elucidating the pattern of influence of ELF-EMF exposure. Data presented in the current study indicates that a range of kinetic constants, such as total activity, rate of increase, decrease in enzymatic activities, peak time and specific activity of enzymes are both amenable to rapid change and influenced by ELF-EMF exposure. Furthermore, key mediators of the inflammatory response, likewise, appear receptive to swift modulation. This is exemplified by an ELF-EMF-induced elevation in the expression of IL-18BP, which provides a signal for terminating the IL-18 mediated inflammatory response, and a rise in TGF-β that can act as an anti-inflammatory cytokine and has shown neuroprotective effects under conditions relevant to AD and following CNS insult [31], [32]. In contrast, 24 hr ELF-EMF exposure insignificantly affected the expression of MCP-1, involved in the neuroinflammatory processes associated with diseases characterized by neuronal degeneration [62], [63], or of IL-18, likewise, a key player in neuroinflammation and degeneration [64], [33], [34].

Albeit our results on cytokine expression, despite differences in experimental conditions, are in line with several ELF-EMF exposure studies [44], they are not in accord with ones reporting ELF-EMF promotion of cellular neurodifferentiation, as exemplified by neurite extension and number [65], [41]. Although our ELF-EMF exposure condition induced an early rise in NOS and O_2_^−^, and adaptive cellular changes in CAT activity, it did not exert any cytotoxic or phenotypic changes, as confirmed by the fact that cell morphology and proliferation were unaltered after 6 and 24 hr ELF-EMF (Fig. 1).

In synopsis, we found SH-SY5Y cells useful in the evaluation of neuronal ELF-EMF mediated actions. An ELF-EMF exposure of 1mT proved well tolerated and without relevant action on cellular survival and metabolism, but significantly impacted the balance of oxidative stress and gene transcription of select inflammatory cytokines. This latter result provides impetus to undertake evaluation of a more complete list of genes that are time-dependently up- and down-regulated by ELF-EMF exposure to elucidate its modulatory actions. Our results suggest that adaptive mechanisms can rapidly offset exposure in unchallenged neuronal cultures to maintain homeostasis. However, confronted with existing oxidative stress, as induced by co-exposure to H_2_O_2_ (Fig. 5), the susceptibility of neuronal cells to free radicals and challenge to maintain homeostasis proved greater. H_2_O_2_-induced stress in cells co-exposed to continuous ELF-EMF proved to be not well counteracted, resulting in a reduction of CAT activity and a rise in O_2_^−^ levels. Hence, in accord with Falone and colleagues [41], contuinuous ELF-EMF exposure reduced cell tolerance towards simultaneous oxidative challenges, and may aid our understanding of the role of concurrent environmental agent challenges in the development of neurodegenerative diseases.

It is important to note, however, that studies by others [66]–[68] have demonstrated that ELF-EMF exposure, in the form of transcranial magnetic stimulation (60-Hz, 0.7 mT) applied to rats for 2 hr twice daily, can prove neuroprotective. Administered prior to and after a toxic insult to the brain, for example systemic injection of 3-nitropropionic acid to induce an animal model of HD [69], ELF-EMF can mitigate oxidative damage, elevate neurotrophic protein levels in brain and ameliorate behavioral deficits [66]–[68], as well as potentially augment neurogenesis [70]. Such studies reiterate that the level and timing of exposure are critical factors impacting outcome measures, and can be potentially scheduled to optimize endogenous compensatory mechanisms following an adverse challenge.

Further studies designed to evaluate the actions of ELF-EMF under multiple conditions, including chronic or sporadic exposure in combination with common stressors pertinent to real life, appear warranted and may both aid our understanding of the true biological impact of ELF-EMF and scientifically anchor proposed exposure limits.
